# Neurotrophin signalling in amygdala-dependent cued fear learning

**DOI:** 10.1007/s00441-020-03260-3

**Published:** 2020-08-26

**Authors:** Susanne Meis, Thomas Endres, Volkmar Lessmann

**Affiliations:** 1 Institut für Physiologie, Medizinische Fakultät, Otto-von-Guericke-Universität, Leipziger Str. 44, 39120 Magdeburg, Germany; 2 Center for Behavioral Brain Sciences, Magdeburg, Germany

**Keywords:** Amygdala, LTP, Fear, Extinction, BDNF, GABA

## Abstract

The amygdala is a central hub for fear learning assessed by Pavlovian fear conditioning. Indeed, the prevailing hypothesis that learning and memory are mediated by changes in synaptic strength was shown most convincingly at thalamic and cortical afferents to the lateral amygdala. The neurotrophin brain-derived neurotrophic factor (BDNF) is known to regulate synaptic plasticity and memory formation in many areas of the mammalian brain including the amygdala, where BDNF signalling via tropomyosin-related kinase B (TrkB) receptors is prominently involved in fear learning. This review updates the current understanding of BDNF/TrkB signalling in the amygdala related to fear learning and extinction. In addition, actions of proBDNF/p75NTR and NGF/TrkA as well as NT-3/TrkC signalling in the amygdala are introduced.

## Introduction

The amygdala is a telencephalic group of diverse, interconnected nuclei in the brain (Pitkanen et al. 1997; Knapska et al. 2007) that has been implicated in a wide variety of functions like emotion, motivation, learning and memory (Aggleton 1993; LeDoux 2000; Seymour and Dolan 2008; Pape and Pare 2010; Tye 2018). This region, located near the temporal pole of the mammalian cerebral hemisphere, is generally divided into several nuclei according to neuroanatomical and cytoarchitectural characteristics (Swanson and Petrovich 1998; Pitkanen et al. 2000; Sah et al. 2003; LeDoux 2007). Among these, the basolateral complex of the amygdala (BLA), which includes the lateral (LA) and basal (BA) nuclei and the central amygdala (CE), divided into the lateral (CEl) and medial (CEm) parts, are critically involved in learning and memory of fearful events as assessed commonly by fear conditioning. In this experimental model, an initially neutral conditioned stimulus (CS), such as a light or tone, is contingently paired with an innately aversive unconditioned stimulus (US), routinely a foot-shock (Fendt and Fanselow 1999; Davis and Whalen 2001; Maren 2001; Maren and Quirk 2004). After a successful association between CS and US during the training phase, the CS can provoke defensive responses like freezing behaviour independently of the US (Rodrigues et al. 2004; Johansen et al. 2011; Tovote et al. 2015; Izquierdo et al. 2016). The neural circuits underlying this form of Pavlovian fear conditioning have been thoroughly investigated (Pape and Pare 2010). According to the prevailing model, the association about CS and US takes place in the LA, where sensory information representing the CS or US, respectively, converge (Sigurdsson et al. 2007). Indeed, damage or functional inactivation of the LA impairs acquisition and expression of fear responses to the CS (Gale et al. 2004; Sigurdsson et al. 2007 and references therein). Subsequently, activation of LA neurons upon CS presentation triggers fear responses by recruitment of CE and its projections to the brain stem and hypothalamus (Fendt and Fanselow 1999; Fanselow and Poulos 2005; Davis and Whalen 2001; Maren 2005; Sigurdsson et al. 2007). Beyond a role in relaying information from LA to downstream effectors, the CE is meanwhile considered as an integral site of plasticity and integration for fear and reward learning (Pare et al. 2004; Ehrlich et al. 2009; Duvarci and Pare 2014; Fadok et al. 2018; Tye 2018). Moreover, recent data demonstrate that fear memory relies on a broadly distributed network, including several brain regions, e.g., hippocampus, cortex and periaqueductal grey (Balleine and Killcross 2006; Ehrlich et al. 2009; Marek et al. 2013; Duvarci and Pare 2014; Herry and Johansen 2014; Yu et al. 2017; Aschauer and Rumpel 2018; Grössl et al. 2018; Headley et al. 2019; Ressler and Maren 2019; Sun et al. 2020).

According to the cellular hypothesis of cued fear learning, CS-US convergence induces associative plasticity in LA projection neurons, leading to enhanced cellular activity upon CS presentation alone (Blair et al. 2001; Sigurdsson et al. 2007; Sah et al. 2008; Pape and Pare 2010; Johansen et al. 2012; Sears et al. 2014). Indeed, neural responses to the CS are enhanced in the LA in vivo and in vitro after fear learning (for review and references, see Schafe et al. 2001; Sigurdsson et al. 2007; Sah et al. 2008; Johansen et al. 2012). In addition, induction of long-term potentiation (LTP) as a cellular model for learning and memory, was described after high-frequency stimulation of auditory afferences to the LA in vivo and in vitro (Blair et al. 2001; Sigurdsson et al. 2007; Sah et al. 2008; Johansen et al. 2012; Sears et al. 2014). In fact, this LTP was occluded in slices prepared from rodents after fear learning (Pape and Pare 2010; Hong et al. 2011; Meis et al. 2018 and references therein). Furthermore, LTP and fear conditioning share numerous molecular mechanisms (Rodrigues et al. 2004; Johansen et al. 2011). Therefore, strong evidence corroborates LTP as a cellular mechanism of fear learning in the amygdala (Sigurdsson et al. 2007; Sah et al. 2008; Luchkina and Bolshakov 2019), thus motivating detailed studies of the physiology and pathophysiology of fear learning.

### Distribution of BDNF/TrkB in the amygdala

Several neurotrophins and their cognate receptors have been identified, with TrkA (tropomyosin-related kinase A) receptors preferentially activated by NGF (nerve growth factor), TrkB receptors activated by BDNF as well as NT-4/5 (neurotrophin-4/5) and TrkC receptors by NT-3 (neurotrophin-3) (Barbacid 1995; Edelmann et al. 2014).

While many findings regarding BDNF/TrkB signalling in cellular aspects of learning were initially reported for hippocampal and cortical circuits, BDNF/TrkB pathways in the amygdala emerged soon thereafter to be prominently involved in fear learning (see below). TrkB receptor as well as BDNF mRNA and protein were detected at moderate to high levels in various amygdala subnuclei (Masana et al. 1993; Altar et al. 1994; Conner et al. 1997; Yan et al. 1997; Krause et al. 2008). Besides BDNF, TrkB receptors can also be activated by NT-4/5. Although expression of NT-4/5 has been reported in the postnatal hippocampus, neocortex, cerebellum and thalamus (Friedman et al. 1998), prominent expression of NT-4/5 in the amygdala has thus far not been reported. Moreover, at variance with BDNF, NT-4/5 is spared from the regulated pathway of secretion (Lessmann and Brigadski 2009). Therefore, BDNF is considered the main ligand of TrkB receptors in the rodent amygdala. This view is corroborated by the finding that BDNF expression was increased in the amygdala after fear conditioning, while NT-4/5 expression remained unchanged (see below).

Sensory information representing the CS and US, respectively, enters the LA from thalamic and cortical regions. Thalamic projections to LA include the medial division of the medial geniculate body (MGm), the posterior intralaminar nucleus (PIN) and the suprageniculate nucleus (SG) (Farb and LeDoux 1997). In addition, MGm and PIN represent areas of acoustic and nociceptive convergence (LeDoux et al. 1987) and may therefore also transmit somatosensory US input to the LA (Pape and Pare 2010). These thalamic neurons exhibit BDNF expression in their soma and fibres (Kawamoto et al. 1996; Conner et al. 1997; Furukawa et al. 1998). Furthermore, auditory information about the CS is transferred to LA by the temporal association cortex (Mascagni et al. 1993; Romanski and LeDoux 1993; Shi and Cassell 1997; McDonald 1998), which also shows substantial BDNF expression (Conner et al. 1997). US inputs to LA are less well described but may include various midline thalamic nuclei and the anterior cingulate cortex (ACC) (Herry and Johansen 2014). In the cingulate cortex, light to moderate BDNF immunoreactivity as well as heavy staining for BDNF mRNA was detected (Kawamoto et al. 1996; Conner et al. 1997; Furukawa et al. 1998). In addition, the pontine parabrachial nucleus and the paraventricular thalamic nucleus were identified as a major source of BDNF for the lateral nucleus of the central amygdala (Conner et al. 1997; Penzo et al. 2015). Accordingly, histochemical evidence supports a prominent role for BDNF/TrkB signalling at pre- and postsynaptic sites within the amygdala.

### Role of amygdala BDNF/TrkB signalling in cued fear learning

Several lines of evidence reveal a substantial contribution of BDNF/TrkB signalling in fear learning (as reviewed by Rattiner et al. 2005; Cowansage et al. 2010; Musumeci and Minichiello 2011; Andero et al. 2014; Ehrlich and Josselyn 2016). After cued fear conditioning, BDNF mRNA was transiently elevated in the rodent BLA, while NGF, NT-4/5 and NT3 expression remained unchanged (Rattiner et al. 2004a; Jones et al. 2007). Specifically, a selective increase in BDNF transcripts containing exons I and III was detected (Rattiner et al. 2004b; Ou and Gean 2007). Upregulation of BDNF expression after fear conditioning required calcium influx, protein kinase A, calcium/calmodulin-dependent kinase IV (CaMKIV) and cAMP response element–binding protein (CREB) phosphorylation (Ou and Gean 2007).

In parallel, levels of BDNF protein as well as TrkB receptor phosphorylation were temporarily increased after fear conditioning in the BLA (Ou and Gean 2006). In particular, fear conditioning resulted in the interaction between TrkB and Shc, followed by a transient increase in Ras bound to Shc and activation of mitogen-activated protein kinase (MAPK) and phosphatidylinositol-3 (PI-3) kinase (Ou and Gean 2006). Recently, a transient increase in BDNF levels after fear conditioning was also demonstrated in mice subjected to chronic social defeat stress. Subsequently, these mice could be classified as ‘susceptible’ or ‘resistant’, according to their social interaction behaviour. Interestingly, susceptible mice showed an elevated increase in BDNF protein in the BLA after fear training as well as elevated cued fear learning compared with resistant mice (Chou et al. 2014). Likewise, a positive correlation between the expression of conditioned fear and amygdala BDNF levels was reported in wild-type mice (Yee et al. 2007; Endres and Lessmann 2012). Therefore, individual BLA BDNF protein levels seem to be related to cued fear learning abilities. In addition, activation of TrkB receptors by application of exogenous BDNF into the BLA or by systemic administration of the TrkB receptor agonist 7,8-dihydroxyflavone (7,8-DHF) enhanced fear learning (Ou and Gean 2006; Andero et al. 2011). Interestingly, proteolytic cleavage of proBDNF by plasmin was required for fear learning (Ou and Gean 2007), supporting a role for mature BDNF in this process. Furthermore, lower levels of mature BDNF were detected in the amygdala and hippocampus of proprotein convertase 7 (PC7) knockout mice, presumably due to reduced proBDNF processing. Indeed, PC7 knockout mice were impaired in cued fear learning and this deficit could be rescued by systemic administration of the TrkB agonist 7,8-DHF (Wetsel et al. 2013).

Importantly, inhibition of BDNF/TrkB signalling in the amygdala impaired fear memory. This was shown by overexpression of a non-functional, truncated TrkB receptor in the BLA, as well as by local BLA infusion of a tyrosine kinase inhibitor or TrkB ligand scavenger, respectively (Rattiner et al. 2004a; Ou and Gean 2006; Ou et al. 2010). In addition, the BDNF protein level increased a second time at 12 h after fear conditioning and this peak in BDNF expression was shown to be required for memory persistence (Ou et al. 2010).

In conclusion, BDNF mRNA expression as well as BDNF protein levels are elevated after cued fear learning. Subsequently, BDNF activates TrkB receptors as indicated by the increased TrkB phosphorylation after fear conditioning. Concurrently, fear learning and BDNF levels are positively correlated. The need for BDNF/TrkB signalling in fear learning is further accentuated by impaired fear memory when BDNF/TrkB signalling is blocked before fear training.

Additional evidence for the critical involvement of BDNF/TrkB signalling in cued fear learning and memory was gained by examination of genetic mouse models. Fear learning was impaired in mice carrying a point mutation in the Y816 (PLCγ) or Y515 (Shc) phosphorylation site of the TrkB receptor, respectively (Musumeci et al. 2009). The PLCγ site seemed to facilitate the acquisition of conditioned fear responses, while the Shc site was mainly involved in memory consolidation (Musumeci et al. 2009). Interestingly, overexpression of TrkB receptors led to selective activation of the TrkB-PLCγ pathway, while cued fear learning remained unaltered when tested 24 h after fear conditioning (Koponen et al. 2004). This may point to a specific role of TrkB-Shc signalling in amygdala-dependent fear learning under these experimental conditions.

Another possibility to study BDNF signalling in behavioural tasks is the use of genetic models that display a reduction in BDNF levels or BDNF secretion. Interestingly, controversial results were reported concerning cued fear learning in distinct transgenic lines. For instance, cued fear learning was impaired or retained depending on the time of induction of forebrain-restricted BDNF knockout mice (Gorski et al. 2003; Monteggia et al. 2004). Mice carrying a point mutation in the BDNF gene (BDNF^Val/Met^ or BDNF^Met/Met^), which drives the expression of Met-BDNF and leads to decreased activity-dependent BDNF secretion (Egan et al. 2003), showed intact cued fear learning (Chen et al. 2006; Soliman et al. 2010). However, cued fear responses in human BDNF Met allele carriers (BDNF^Val/Met^ or BDNF^Met/Met^) were unaffected or impaired when assessed by skin conductance response or fear-potentiated startle, respectively (Chen et al. 2006; Soliman et al. 2010; Lonsdorf et al. 2010). Previous studies reported intact cued fear learning in heterozygous BDNF^+/−^ mice (Liu et al. 2004; Chen et al. 2006; Karpova et al. 2011) while reduced conditioned fear responses were observed in BDNF^+/−^ rats (Harris et al. 2016). Moreover, we could show that young adult BDNF^+/−^ mice develop an age-dependent fear learning deficit starting at 3 months postnatally, which could be attributed to a deficit in memory consolidation (Endres and Lessmann 2012; Meis et al. 2018). This learning deficit was rescued when a stronger fear conditioning protocol was executed (Endres and Lessmann 2012). Taken together, the impact of chronic BDNF depletion on cued fear learning is highly variable. This may relate to delayed and/or altered development and compensatory mechanisms under conditions of reduced BDNF availability, which may rely on the extent, time and region of BDNF depletion. Furthermore, a critical threshold level of BDNF may be required for fear processing through modulation of synaptic plasticity (see e.g., Korte et al. 1995), which may in turn depend on species, age or the employed behavioural approach, respectively.

Another important aspect to evaluate the above-mentioned discrepancies is the fact that adult BDNF^+/−^ mice, albeit showing intact short-term memory, displayed a continuous loss in fear memory precision with ongoing time after fear training. Moreover, these BDNF^+/−^ mice were unable to discriminate tones associated with foot-shock (CS+) from tones not paired with the aversive US (CS−) in a discriminative fear learning task (Meis et al. 2018). In line with these observations, human Met allele carriers took longer to recognize that the neutral cue was not associated with an aversive stimulus (Soliman et al. 2010) and showed enhanced generalization of cued fear to a novel context (Muhlberger et al. 2014). Diminished fear specificity was also recognized in forebrain-restricted BDNF knockout mice, which showed enhanced freezing prior to presentation of the CS (pre-CS) with similar freezing levels to pre-CS or CS, respectively (Gorski et al. 2003). Correspondingly, a fear-generalized mouse model displayed lower levels of BDNF in the BLA immediately after fear conditioning with high US intensity, which lasted at least 2 weeks (Asim et al. 2020). Thus, several lines of evidence support a significant role of BDNF/TrkB signalling in memory precision and cued fear discrimination.

### Cellular mechanisms of amygdala BDNF/TrkB signalling in cued fear learning

**Fig. 1:**
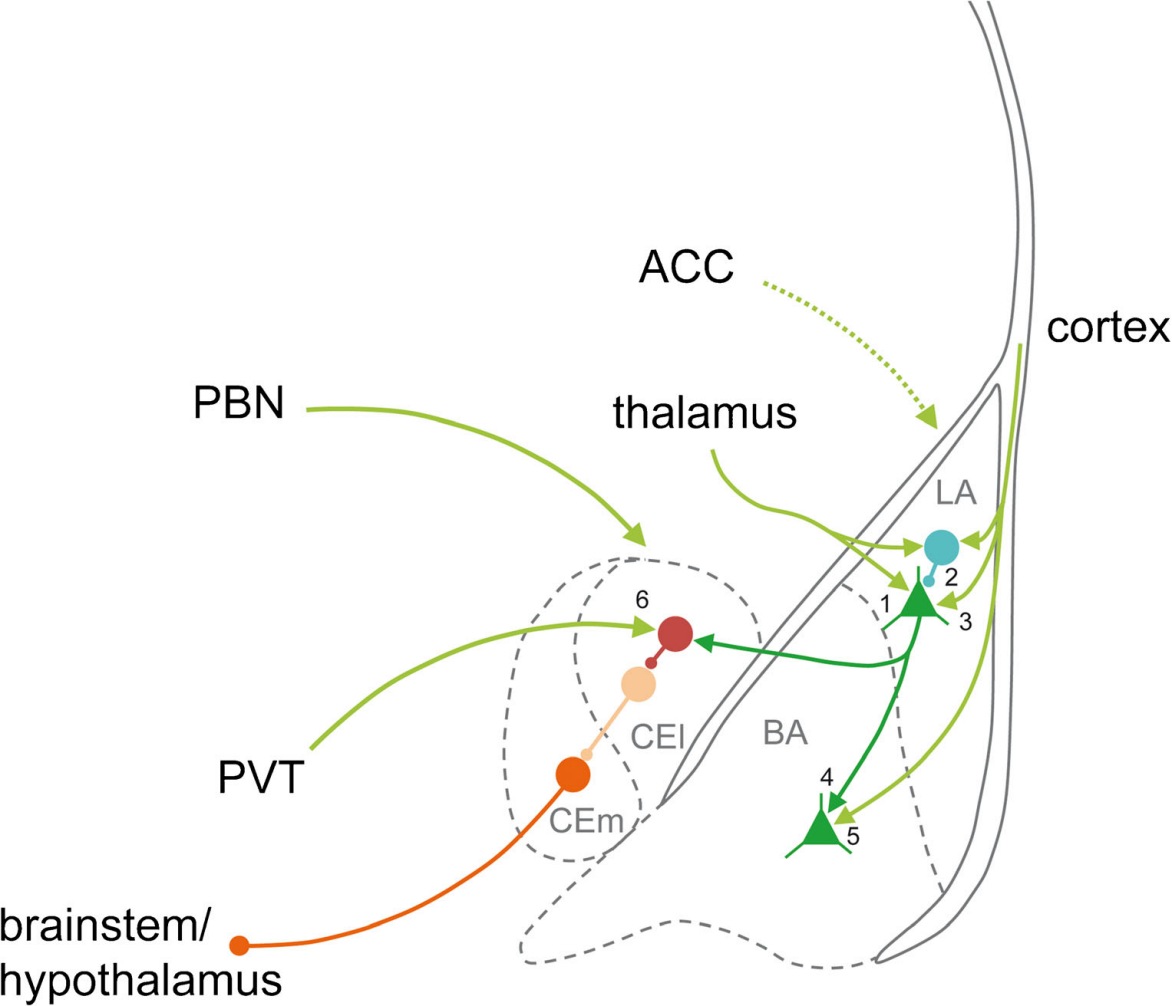
Cellular actions of BDNF in the amygdala. Schematic representation of afferents to different subnuclei of the amygdala that probably contain BDNF (green). Numbers depict cellular effects of BDNF/TrkB signalling on synaptic plasticity/cellular activity shown for the respective synapse. 1: Meis et al. 2012; Musumeci et al. 2009, 2: Mou et al. 2011, 3: Meis et al. 2018, 4: Musumeci et al. 2009, 5: Li et al. 2011, 6: Penzo et al. 2015. ACC: anterior cingulate cortex, PVT: paraventricular thalamic nucleus, PBN: parabrachial nucleus (modified from Paxinos and Franklin 2001)

Several studies support the notion that BDNF/TrkB signalling contributes significantly to cue-dependent fear learning by enhancing synaptic plasticity in the amygdala. Specifically, in vitro recordings revealed support of LTP by BDNF/TrkB signalling at thalamic and cortical afferents to LA (see Fig. 1). These projections transmit convergent sensory information about the CS and US to the LA and are as thus essential for fear learning (Sigurdsson et al. 2007; Pape and Pare 2010; Johansen et al. 2012). Specifically, LTP was blocked by application of a scavenger for endogenous BDNF (i.e., TrkB receptor bodies) at thalamic afferents to LA, as assessed by whole-cell recordings from LA projection neurons. Moreover, inclusion of the tyrosine kinase inhibitor K252a in the pipette solution prevented the induction of LTP, which suggests a postsynaptic site of action of BDNF in mediating plasticity at this synaptic input (Meis et al. 2012). Application of K252a also abolished LTP induction at cortico-LA synapses in field potential recordings obtained from slices of adult mice (Meis et al. 2018). In line with these results, field potential recordings demonstrated impaired LTP at the thalamic input to LA in mice carrying point mutations at either the Shc or the PLCγ docking site of TrkB (Musumeci et al. 2009).

Interestingly, chronic BDNF reduction to about 50% of wild-type levels in BDNF^+/−^ mice lead to impaired LTP at thalamic afferents to LA, whereas LTP at the cortical input to LA was unaffected. However, intact cortico-LA LTP was present at 3 months of age and beyond, when a fear memory consolidation deficit was observed in BDNF^+/−^ mice (Endres and Lessmann 2012; Meis et al. 2012, 2018). In wild-type mice, fear learning induced consolidation-relevant synaptic plasticity at cortico-LA synapses in vivo, which occluded induction of ex vivo LTP at 4 and 24 h after training. These long-term changes as well as occlusion of LTP were absent in BDNF^+/−^ mice, which did not show intact memory consolidation. Therefore, synaptic plasticity as a prerequisite for fear memory consolidation, which might take place inside and outside of the amygdala, seems to be absent in young adult BDNF^+/−^ mice (3–4 months of age) (Meis et al. 2018).

The LA is highly interconnected with the BA (Pitkanen et al. 1997), which is also implicated in fear learning (Amano et al. 2011). Consistently, synaptic plasticity at LA–BL synapses was impaired in TrkB/PLCγ mutant mice (Musumeci et al. 2009). At cortical inputs to BA, LTP was inhibited by application of the BDNF scavenger TrkB-IgG, while administration of exogenous BDNF or 7,8-DHF facilitated LTP induction in this pathway (Li et al. 2011). In line with these results, chronic treatment with 7,8-DHF was reported to enhance the activation of phosphorylated TrkB at the Y515 and Y816 sites. Concurrently, synaptic plasticity in the basolateral amygdala was facilitated and age-related declines in fear learning were prevented in rats at the age of 25 months (Zeng et al. 2012).

Beside glutamatergic synaptic transmission, BDNF/TrkB signalling also regulates GABAergic neurotransmission (Gottmann et al. 2009), which closely controls excitatory circuits in the amygdala and thereby critically regulates fear learning (Pare et al. 2003; Ehrlich et al. 2009; Duvarci and Pare 2014; Letzkus et al. 2015; Krabbe et al. 2018; Lucas and Clem 2018). In particular, BDNF/TrkB signalling was shown to induce rapid internalization of GABA_A_Rα1 subunits in amygdala cell cultures, which was supposed to elicit a transient hyper-excitability in the amygdala, thereby contributing to cellular mechanisms of memory consolidation (Mou et al. 2011, 2013). Indeed, many findings support a pivotal role for disinhibition of principal LA neurons as a permissive factor in fear conditioning (Duvarci and Pare 2014). Nevertheless, BDNF^+/−^ mice displayed neither impaired basal synaptic GABAergic transmission nor altered inhibitory synaptic plasticity in the LA. However, positive modulation of interneuron activity by noradrenaline was significantly decreased by chronic BDNF reduction (Meis et al. 2019). In line with these results, BDNF depletion also abolished facilitation of synaptic GABAergic transmission by serotonin (Daftary et al. 2012). In conclusion, BDNF signalling may contribute directly as well as indirectly (i.e., via altered neuromodulation) to the regulation of inhibitory synaptic circuits in the amygdala. Transient disinhibition may facilitate fear learning, while a chronic BDNF deficit might destabilize the balance between inhibition and excitation and impair amygdala function.

Beside the basolateral amygdala, the central nucleus is now considered as an important site of associative plasticity involved in fear memory (Ehrlich et al. 2009; Fadok et al. 2018). Recent evidence indicates a significant role of the paraventricular nucleus of the thalamus (PVT) in fear memory consolidation and retrieval (Arruda-Carvalho and Clem 2015; Do Monte et al. 2016). Consistent with the expression of BDNF in the PVT (Conner et al. 1997), relevance of BDNF/TrkB signalling in the PVT/CE circuitry was detected (Penzo et al. 2015). Specifically, selective deletion of either BDNF expression in the PVT or TrkB receptors in CEl neurons similarly impaired fear learning, while infusion of BDNF into the CEl enhanced fear learning and elicited unconditioned fear responses (Penzo et al. 2015). At the cellular level, absence of TrkB receptors in somatostatin-positive CEl neurons prevented the fear conditioning–induced strengthening of excitatory synaptic transmission in these cells, while bath application of BDNF markedly increased their spiking probability in vitro (for details, see Penzo et al. 2015). These BDNF/TrkB mediated changes seem to appear parallel to dopamine-dependent changes in CEl synaptic transmission (Groessl et al. 2018) highlighting the importance of fine tuning between BDNF and neuromodulator signalling in shaping fear memories. Moreover, these results emphasize the critical contribution of BDNF/TrkB signalling in distinct subnuclei of the amygdala during fear processing.

In conclusion, BDNF/TrkB signalling increases excitatory synaptic transmission in different subnuclei of the amygdala and enables LTP. At GABAergic synapses, acute BDNF/TrkB signalling may lead to reduced inhibition and elevated excitability necessary for memory formation, while chronic BDNF reduction results in impaired interaction of GABAergic synaptic transmission with modulatory transmitters like noradrenaline and serotonin.

### Amygdala BDNF/TrkB signalling and cued fear extinction

**Fig. 2:**
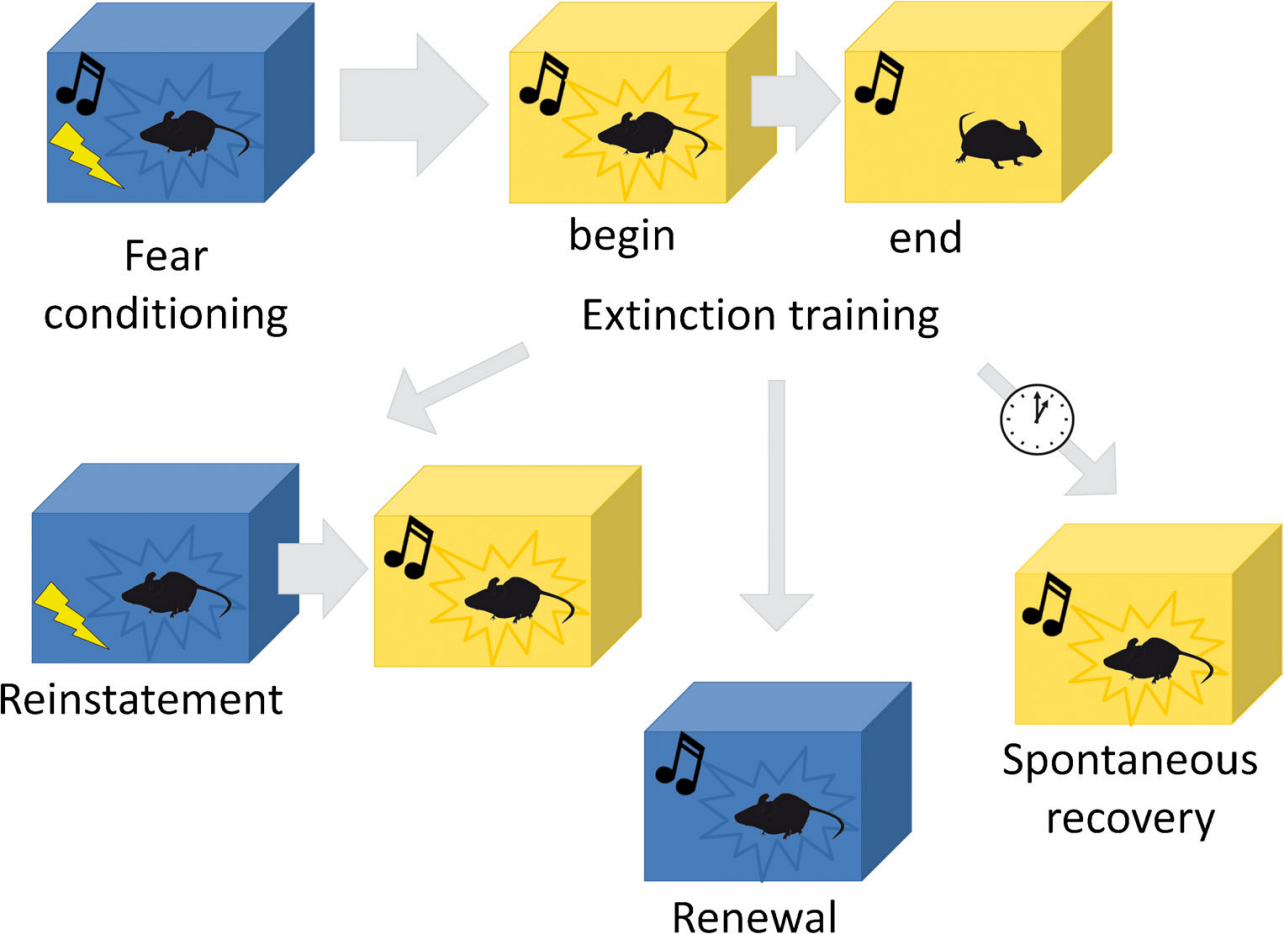
Simplified scheme of cued fear extinction learning. Relapse of extinguished fear may occur after exposure to the US (reinstatement), in contexts different from the extinction training context, for example the original fear context (renewal), or with passing of time (spontaneous recovery). Freezing is depicted as mouse surrounded by star-shaped edges. Different colours represent different contexts

After fear learning, repeated exposure to the conditioned stimulus alone leads to diminished fear responses (Myers and Davis 2007; Pape and Pare 2010; Milad and Quirk 2012; Singewald and Holmes 2019; Sangha et al. 2020). This process, termed fear extinction learning (see Fig. 2), involves several brain areas, particularly the amygdala, hippocampus and the medial prefrontal cortex (PFC) (Singewald et al. 2015; Maren and Holmes 2016; Singewald and Holmes 2019; Marek et al. 2019). Extinguished fear may reappear after exposure to the US (reinstatement), in contexts different from the context where extinction training took place (renewal), or with passing of time (spontaneous recovery) (Singewald and Holmes 2019). Therefore, it is generally accepted that fear extinction relies on the generation of a new inhibitory memory that actively supresses the original one. Several studies established the prominent role of intercalated inhibitory amygdala neurons and the infralimbic mPFC in this process (Pape and Pare 2010; Duvarci and Pare 2014). Additionally, recent observations support the notion that alterations induced by fear learning may be reversed by extinction learning (Quirk et al. 2010; Herry et al. 2010; Sangha 2015; An et al. 2017).

A critical contribution of BDNF signalling in extinction was recently demonstrated. BDNF^+/−^ mice display an age-dependent deficit in extinction learning (Psotta et al. 2013). Similarly, BDNF^Val/Met^ or BDNF^Met/Met^ mice as well as human Met allele carriers were impaired in extinguishing a conditioned fear response, associated with abnormal fronto-amygdala activity in humans (Soliman et al. 2010). In line with these results, systemic injection of 7,8-DHF promoted extinction learning (Andero et al. 2011). Ample evidence supports the prominent role of BDNF signalling in the hippocampus and infralimbic PFC in the formation of fear extinction memories (Heldt et al. 2007; Peters et al. 2010; Rosas-Vidal et al. 2014; Singewald et al. 2015), with downstream effects on BDNF signalling occurring in the amygdala. BDNF protein levels were initially increased in the ventral hippocampus after extinction training, preceding extinction-induced expression of BDNF in the BA (Rosas-Vidal et al. 2014). Thus, BDNF signalling was suggested to be recruited subsequently by afferents from the ventral hippocampus to the BA. Inhibition of BDNF/TrkB signalling by infusion of a dominant-negative TrkB receptor into the BLA prior to extinction training impaired retention of extinction memory (Chhatwal et al. 2006). Interestingly, chronic treatment with the antidepressant drug fluoxetine resulted in enduring loss of conditioned fear memory when combined with extinction training in adult mice (Karpova et al. 2011). In parallel, BDNF mRNA levels were increased in the amygdala and hippocampus, suggesting that BDNF signalling in the BLA is critically involved in fear extinction. Fear erasure, measured as impaired fear renewal after extinction learning, was not observed in fluoxetine treated BDNF^+/−^ mice but was mimicked by overexpression of BDNF in the BLA from the end of extinction training onward (Karpova et al. 2011). It was suggested that fluoxetine treatment reactivates juvenile-like plasticity in the amygdala through local BDNF action (Karpova et al. 2011; Umemori et al. 2018). Similarly, combining administration of an amphetamine derivative (3,4-methylenedioxymethamphetamine, ‘ecstasy’) with extinction training, significantly increased BDNF expression in the amygdala and facilitated fear extinction memory induced by a suboptimal training paradigm (Young et al. 2015). Interruption of BDNF signalling in the basolateral complex by local infusion of a BDNF neutralizing antibody blocked this enhancing effect on fear extinction by amphetamine treatment (Young et al. 2015). Besides facilitation of fear extinction memory by BDNF actions in the amygdala, BLA TrkB receptors situated at BLA afferents presynaptic to the infralimbic PFC were reported to facilitate fear extinction memory (Li et al. 2017). Overall, evidence indicates significant contribution of BDNF signalling in the amygdala to fear extinction memory, while the underlying BDNF/TrkB regulated cellular mechanisms are thus far poorly resolved.

### ProBDNF/p75NTR signalling in the amygdala

Neurotrophins are at first synthesized as precursor proteins, which are processed to the mature form by proteolytic cleavage (Lessmann and Brigadski 2009). As recently recognized, pro-neurotrophins, the cleaved pro-domain as well as mature neurotrophins exert distinct cellular functions (compare Brigadski et al., Kojima et al. in this issue). Specifically, pro-neurotrophins interact with the p75 neurotrophin receptor (p75NTR) and often induce effects that oppose those of mature neurotrophins when binding to their cognate Trk receptors (for reviews, see Costa et al. 2018; Sasi et al. 2017; Zanin et al. 2017; Gibon and Barker 2017; Notaras and van den Buuse 2018; Becker et al. 2018; Von Bohlen und Halbach and Von Bohlen und Halbach 2018). In line with the role of p75NTR signalling in apoptosis during neuronal differentiation, p75NTR is widely expressed in the developing nervous system and is substantially downregulated in adulthood (Roux and Barker 2002; Underwood and Coulson 2008; Ibanez and Simi 2012; Foltran and Diaz 2016). Immunoblotting revealed the presence of p75NTR in the adult murine amygdala (Algamal et al. 2018; Colyn et al. 2019) while a lack of p75NTR expression was detected by immunohistochemistry (Giza et al. 2018). These seemingly contradictory findings might reflect the complex technical requirements for the detection of low p75NTR expression levels in the adult brain (as suggested by Baho et al. 2019). Interestingly, constitutive p75NTR knockout mice showed augmented cholinergic innervation of the amygdala (Busch et al. 2017). In addition, the dopaminergic and serotoninergic transmitter system as well as synaptic plasticity within this structure were altered (Busch et al. 2017). However, p75NTR knockout mice displayed equal fear responses as their wild-type littermates in contextual or cued fear learning paradigms (Busch et al. 2017). These results are in line with the requirement of proteolytic cleavage of proBDNF to mature BDNF in the amygdala during fear learning, as well as the consolidation of defeat-related memories, indicating the prominent role of mature BDNF in aversive learning (Ou and Gean 2007; Dulka et al. 2016). However, chronic stress exposure was reported to modulate the proBDNF/p75NTR system. Specifically, the levels of p75NTR as well as proBDNF were significantly reduced in amygdala lysates when tested directly after repeated unpredictable stress (Algamal et al. 2018). In contrast, chronic social defeat stress led to enhanced proBDNF expression in the BLA following an aversive social stimulus 1 month afterwards (Colyn et al. 2019). Thus, stress appears to be accompanied by an acute and long-lasting imbalance in the regulation of proBDNF/p75NTR expression in the amygdala. Overall, proBDNF/p75NTR-signalling seems to be still functional in the adult amygdala. However, the actions of its signalling pathways in amygdala circuits remain unresolved.

### Role of other neurotrophins in amygdala function

Conflicting results are available about the distribution of TrkA receptors in the amygdala. While initially neither TrkA receptor mRNA nor immunoreactive cells for TrkA receptors were detected (Gibbs and Pfaff 1994; Sobreviela et al. 1994, 1998), more recently, moderate to strong expression levels were described in the medial and central amygdala (Badowska-Szalewska et al. 2006). The latter study reported regulation of TrkA and NGF immunoreactivity by chronic or acute stress, respectively (Badowska-Szalewska et al. 2006). Labelled NGF-positive cells as well as TrkA receptors were also detected in the BLA (Conti et al. 2009). Both NGF and TrkA protein expression was elevated after chronic exposure to brief non-injurious seizures evoked by minimal electroconvulsive shock and may thereby contribute to neuroprotective effects of NGF (Conti et al. 2009). Likewise, reward sensitization resulted in upregulation of NGF mRNA expression in the CE (Bie et al. 2012), while stress treatment or maternal deprivation, respectively, was followed by reduced NGF content in the amygdala (Lang et al. 2004; Reus et al. 2011). In conditional knockout mice lacking TrkA receptors, amygdala-dependent learning tasks were reported to be intact in young and intermediate-aged mice (Muller et al. 2012), while a forebrain-specific conditional TrkA knockout mouse line displayed impaired cued fear learning (Sanchez-Ortiz et al. 2012). These conflicting results were attributed to the extent of dysfunction of the cholinergic circuitry, differences in mouse genetic background or distinct properties of different Cre lines used in the two studies, respectively (as discussed by Muller et al. 2012). Therefore, NGF/TrkA signalling in the amygdala seems to be linked to stress, reward and neuroprotection, while amygdala-dependent learning may be affected indirectly by NGF/TrkA function during development of cholinergic fibres from the basal forebrain, which constitutes the main cholinergic input to the amygdala.

While neither NT-3 mRNA nor immunoreactive neurons were found in the amygdala (Phillips et al. 1990; Krause et al. 2008), cells positive for TrkC mRNA are widely distributed in LA, BA or CE, respectively, showing intermediate expression levels (Altar et al. 1994; Hassink et al. 1999; Dierssen et al. 2006; Krause et al. 2008). In addition, NT-3 protein was detected in homogenized amygdala tissue (Yee et al. 2007; Reus et al. 2011; Yamada-Goto et al. 2012). TrkC signalling might be essential for maturation and synaptogenesis of amygdala neuronal circuitry during postnatal development (as discussed by Krause et al. 2008) and the maintenance of neuronal integrity during ageing in the amygdala (von Bohlen und Halbach et al. 2003). In addition, TrkC was recently related to synaptic organization and fine tuning of neural connectivity (Naito et al. 2017). In the amygdala, NT-3 content was modulated by maternal deprivation and diet-induced obesity but did not correlate with the expression of conditioned fear (Yee et al. 2007; Reus et al. 2011; Yamada-Goto et al. 2012). Overall, the physiological relevance of NT-3/TrkC signalling in the amygdala is not completely understood (Krause et al. 2008). Remarkably, amygdala function may be indirectly regulated by NT-3/TrkC signalling subsequently to trophic effects of NT-3 on noradrenergic neurons in the locus coeruleus (Dierssen et al. 2006).

## Conclusions

Accumulating evidence indicates that BDNF/TrkB signalling in the amygdala plays a pivotal role in fear learning and memory as well as fear extinction. In the amygdala circuitry, BDNF/TrkB signalling contributes significantly to synaptic plasticity, which is widely accepted as a cellular mechanism underlying fear memory learning. In addition, downstream molecular signalling pathways triggered by TrkB activation are well documented. However, actions of BDNF/TrkB signalling in amygdala synaptic processes involved in fear extinction learning are far less understood. While behavioural studies suggest a significant contribution of BDNF signalling within the amygdala in extinction learning, analysis of the underlying cellular mechanisms warrants further studies.

## Abbreviations

7,8-DHF: 7,8-Dihydroxyflavone
ACC: Anterior cingulate cortex
BA: Basal amygdala
BDNF: Brain-derived neurotrophic factor
BLA: Basolateral amygdala
CaMKIV: Calcium/calmodulin-dependent kinase IV
CE: Central amygdala
CEl: Lateral central amygdala
CEm: Medial central amygdala
CREB: cAMP response element–binding protein
CS: Conditioned stimulus
GABA: Gamma-aminobutyric acid
LA: Lateral amygdala
LTP: Long-term potentiation
MAPK: Mitogen-activated protein kinase
MGm: Medial geniculate body
mPFC: Medial prefrontal cortex
NT-3: Neurotrophin-3
NT-4/5: Neurotrophin-4/5
PC7: Proprotein convertase 7
PI-3 kinase: Phosphatidylinositol-3 kinase
PIN: Posterior intralaminar nucleus
PLCγ: Phospholipase C gamma
PVT: Paraventricular thalamic nucleus
SG: Suprageniculate nucleus
Shc: Specific adaptor protein
Ras: Specific small GTPase
TrkA: Tropomyosin-related kinase A
TrkB: Tropomyosin-related kinase B
TrkC: Tropomyosin-related kinase C
US: Unconditioned stimulus
